# Trends in greenspace use, socioeconomic inequalities and mental health benefits following COVID-19 from 2020 to 2025: a four-wave repeat cross-sectional study of UK adults

**DOI:** 10.1136/bmjph-2025-003311

**Published:** 2026-09-04

**Authors:** Jonathan R Olsen, Natalie Nicholls, Hannah Burnett, Avril Johnstone, Richard Mitchell

**Affiliations:** 1 Institute for Social Science Research, The University of Queensland, Brisbane, Queensland, Australia; 2 School of Health and Wellbeing, University of Glasgow, Glasgow, UK; 3 Faculty of Epidemiology and Population Health, London School of Hygiene & Tropical Medicine, London, UK; 4 Department of Public Health, Policy & Systems, University of Liverpool, Liverpool, UK

**Keywords:** Mental Health, COVID-19, Epidemiologic Factors, Population Surveillance, Public Health

## Abstract

**Introduction:**

Greenspace use provides significant public health benefits and reduces health inequalities. The COVID-19 pandemic precipitated complex shifts in greenspace use patterns and associated socioeconomic inequalities. Monitoring post-COVID-19 greenspace usage patterns is essential to identify persistent inequalities, inform public health monitoring, guide urban development and support pandemic planning. The aim of this study was to examine socioeconomic patterns in greenspace use and its subsequent mental health benefits 5 years post-COVID-19 onset.

**Methods:**

This study employed a four-wave repeat cross-sectional design sampling of 8646 UK adults across distinct time points (April 2020, November 2020, April 2021 and April 2025). Participants reported recent greenspace use, perceived mental health benefits, and demographic characteristics. Logistic regression models adjusting for age, gender, social grade and ethnicity were conducted, with survey weights applied to ensure national representativeness.

**Results:**

Greenspace use in the previous 4 weeks demonstrated consistent upward trajectory from initial lockdown through postpandemic recovery (50% in April 2020, 66% by April 2021, reaching 85% by April 2025). Socioeconomic disparities in use persisted throughout this period, with wave 4 predicted probabilities of 90% for higher versus 80% for lower social grade participants. However, perceived mental health benefits remained equal across socioeconomic strata, with approximately 87% of users reporting mental health benefits from greenspace use.

**Conclusions:**

Greenspace use demonstrated robust sustained growth following the initial pandemic phase, persisting through 5-year follow-up. The majority of users consistently acknowledge mental health benefits from greenspace use. These findings highlight the ongoing value of greenspace for mental well-being and underscore its relevance for urban planning and public health policy. While perceived benefits were equitable, usage disparities remain, warranting targeted strategies to promote access among underrepresented groups. Addressing these inequalities is essential to improve population health and advance UN Sustainable Development Goal 11, which promotes universal access to safe, inclusive and accessible greenspaces.

**WHAT IS ALREADY KNOWN ON THIS TOPIC**

- The COVID-19 pandemic highlighted mental health benefits associated with greenspace use, but revealed usage inequalities; however, post-pandemic greenspace utilisation trends remain unexplored.

**WHAT THIS STUDY ADDS**

- Four-wave nationally representative data (2020–2025) reveals consistent increases in greenspace use, reaching 85% by April 2025; however, socioeconomic inequalities persist, with lower social grades using greenspaces less frequently.
- Mental health benefits from greenspace use are equivalent across all socioeconomic groups and findings demonstrate need for targeted interventions addressing utilisation disparities.

**HOW THIS STUDY MIGHT AFFECT RESEARCH, PRACTICE OR POLICY**

- Greenspace investment and protection remain essential despite constrained public finances and focus on construction-led developments.
- Policymakers must prioritise equitable greenspace provision and access to maximise population mental health benefits.

## Introduction

 Extensive evidence highlights the benefits of greenspace (defined as urban parks, public open spaces, open land with natural vegetation, as well as street trees and greenery) across various health outcomes, including reduced mortality, lower cardiovascular disease risk and improved self-reported general and mental health.^1–5^ These benefits were reinforced in an umbrella review of 40 systematic reviews on greenspace and health.^6^ The mechanisms through which greenspace supports health are well-documented and include mitigation of environmental harms (eg, air and noise pollution), physiological and psychological restoration, promotion of health-related behaviours such as physical activity and enhancement of social well-being.^7–10^ As such, the UN Sustainable Development Goal 11 aims to provide ‘universal access to safe, inclusive and accessible, green and public spaces’ for sustainable cities that allow populations to survive and prosper.^11^

The availability and use of greenspace are shaped by both individual and area-level socioeconomic deprivation. Evidence on access to greenspace in relation to area-level deprivation is mixed; while some studies in the UK suggest that the most deprived areas often have greater local greenspace availability,^12,13^ the total area and quality of these spaces tend to be poorer.^13,14^ However, other global evidence, from Canada,^15^ Germany,^16^ Australia^17^ and the USA,^18^ for example, highlight that more deprived areas and areas with higher marginalised and ethnic population concentrations have poorer access to greenspace. Additionally, individuals of lower socioeconomic position report lower greenspace use compared with their more affluent counterparts,^19,20^ potentially limiting the associated health benefits for those who may gain the most health benefit.^5^ Barriers to greenspace use are multifaceted and include personal factors (eg, age, occupation, motivation), social factors (eg, safety, community), physical factors of the greenspace itself (eg, area, aesthetic, maintenance and human traffic) and structural factors (eg, greenspace policies, discrimination and marginalisation).^21^

The SARS-CoV-2 (COVID-19) pandemic, which emerged in early 2020, led to widespread disruptions in daily life as governments worldwide implemented movement restrictions and ‘stay-at-home’ or ‘lockdown’ orders.^22^ In some countries, such as the UK, businesses, schools, offices and recreational facilities were closed, while many outdoor amenities, including parks and greenspaces, remained accessible, although with time-based restrictions on use.^23^ During this period, greenspace use increased for countries with a higher Gross Domestic Product (GDP),^24^ such as the UK^25^ and Australia,^26^ and decreased in countries with a lower GDP and the most stringent lockdown policies that restricted access to outdoor spaces.^24,27^ Notably, greenspace use saw a sustained rise in the following year^28^ and many of those who accessed greenspaces during the pandemic reported perceived mental health benefits.^25,29,30^

The COVID-19 pandemic had complex and varied effects on inequalities in greenspace use. Some studies found that individuals from the lowest socioeconomic groups experienced the greatest increase in greenspace use compared with those from higher socioeconomic backgrounds.^31^ Conversely, other studies indicated a widening of inequalities, with those from higher socioeconomic positions increasing their greenspace use at a greater rate.^25^ A systematic review of global disparities in greenspace use during the pandemic found differences in greenspace use by income, ethnicity, gender and age.^24^ Reasons for differences between groups were limited access (non-white communities), reduced time-based opportunities (lower income individuals), and feeling vulnerable and anxious about virus transmission (females and older individuals).

Lack of time is frequently reported as a key barrier to greenspace use,^32–34^ and during the COVID-19 pandemic some population groups had reduced time spent travelling to work and out of home, and increased time for socialising, relaxing and leisure.^35^ Though this pattern was socioeconomically patterned where those of lower socioeconomic positions were less likely to be able to work from home.^36^ Behaviours, and subsequent time availability that is associated with greenspace use, have continued to evolve beyond the initial period of the pandemic. In the UK, for example, average daily travel time has increased by 1 hour between March 2020 and March 2024, while free time for socialising has decreased by 50 min. Despite these shifts, time spent on exercise, sports and well-being has remained stable at an average of 20 min per day.^37^ Some prepandemic behaviours have yet to return to previous levels; for instance, bus and rail travel in the UK remain at approximately 90% of 2019 levels, whereas motorised travel has returned to prepandemic pattern.^38^ It is important to examine whether reported greenspace use, perceived mental health benefits and subsequent inequalities have changed in the postpandemic period. This information is important to identify persistent inequalities in reported use and its associated mental health benefits, inform public health monitoring, guide healthy urban development, support future pandemic planning and monitor progress to achieving equitable use of greenspace, as stated in the UN Sustainable Development Goal 11.

The aim of this study is to examine socioeconomic patterns in greenspace use and its subsequent mental health benefits 5 years after the onset of the COVID-19 pandemic.

## Materials and methods

### Study design and data collection

**Figure 1:**
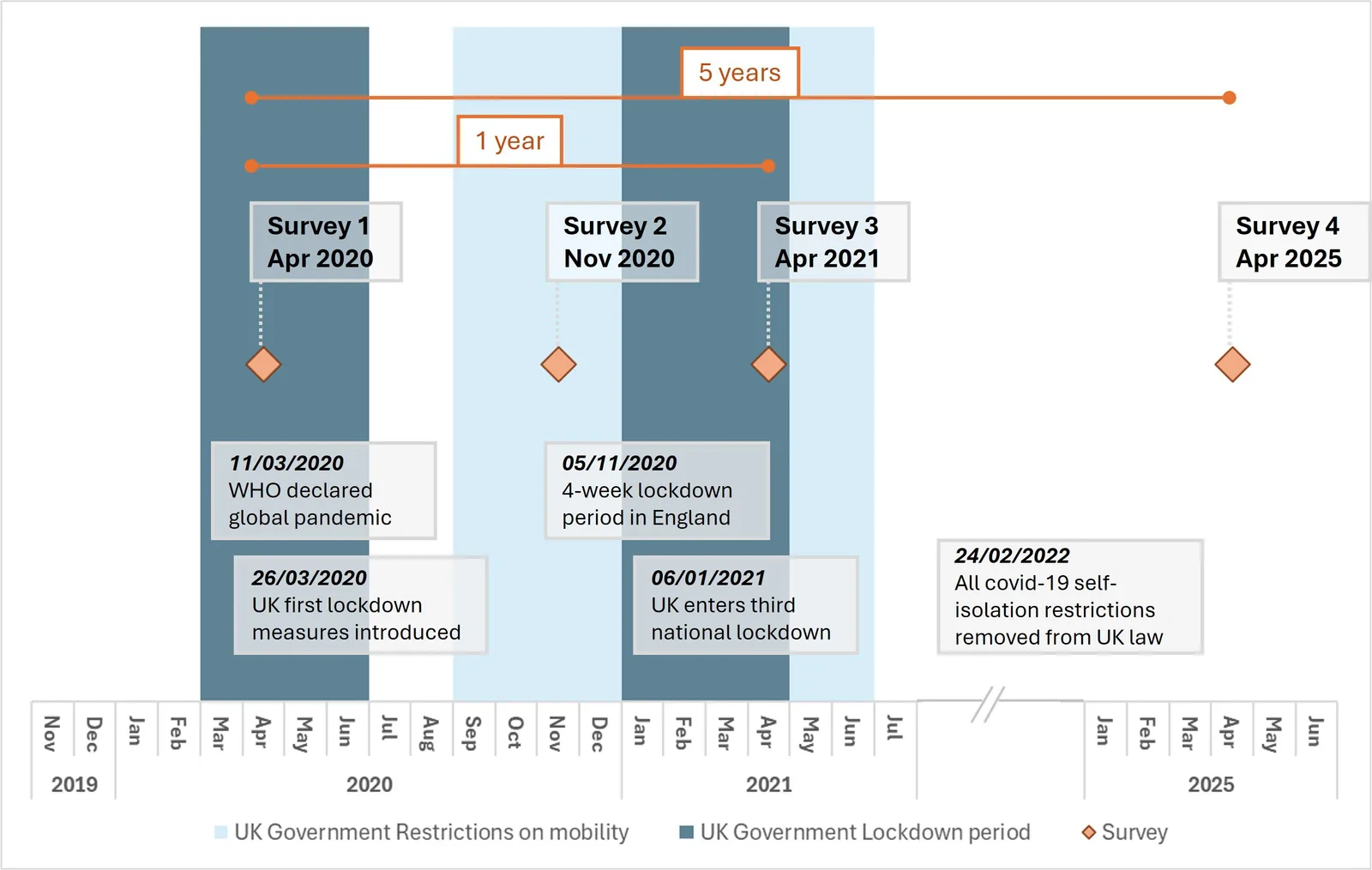
Survey data collection timeline and summary of key UK COVID-19 lockdowns.

This study used primary data collected in the UK across four distinct waves spanning from April 2020 to April 2025 (figure 1) in a repeat cross-sectional study design. Each wave represents a nationally representative cross-sectional sample of the UK population when appropriate weightings were applied.

The survey was designed to align with pre-existing surveys, such as Monitoring Engagement with the Natural Environment survey by Natural England, particularly for the use of green space and barriers to green space questions and responses.^39^ This allowed for direct comparison in outcomes with other national surveys, as well as ensuring validated and acceptable questions were used. With the first wave of the survey being created in early 2020, at the start of the COVID-19 pandemic, there were no existing surveys to review that asked about the impact of the pandemic on green space use. Therefore, the COVID-19-related barriers to green space use were derived from the restrictions on movement (ie, being asked to stay at home), topic experts from the Public Health Scotland Environment and Open Spaces Cross-Sector working group and academic subject experts.

Data were collected by two UK panel survey providers (YouGov and SAVANTA) using the same survey questions, data collection methods and drawn from their UK Omnibus panels, employing a random selection of respondents who received links to complete the survey online. Participants were randomly selected from YouGov and SAVANTA’s UK Omnibus panel and received email invitations with survey links, employing quota sampling methodology.^40^ Informed consent was obtained prior to participating in the UK Omnibus panel for each survey. Data were analysed both by individual wave and combined for cross-wave comparative analyses.

#### April 2020–April 2021 data collection (waves 1–3)

Wave 1–3 data have been previously described in detail,^25,28^ the survey was administered by YouGov and included adults aged ≥18 years. The data collection occurred at various phases of the COVID-19 pandemic: wave 1 (30 April–1 May 2020, 5 years prior to wave 4), wave 2 (26 November 2020) and wave 3 (29 April–30 April 2021, 4 years prior to wave 4), as illustrated in figure 1.

#### April 2025 data collection (wave 4)

The wave 4 survey was administered by SAVANTA in April 2025 (26 April–27 April). The survey included adults (≥16 years) from across the four UK nations (England: n=1788; Scotland: n=180; Wales: n=104; Northern Ireland: n=64).

### Survey measures

#### Demographic variables

The survey collected key demographic and socioeconomic variables known to correlate with greenspace visitation patterns. These included gender (categorised as male, female or prefer not to say), age group (stratified as 16–24, 25–64 and 65+ years), ethnicity (classified as White or Black, Asian and Minority Ethnic (BAME) or other) and social grade (dichotomised into higher: ABC1 and lower: C2DE). Social grade classification adhered to the standard occupational classification, incorporating retired or unemployed individuals, developed by the National Readership Survey (NRS)^41^ serving as a proxy measure for socioeconomic position.

#### Greenspace use

Participants across all waves reported their greenspace visiting frequency during the 4-week period preceding survey. Greenspaces were operationally defined as ‘places where you can see and experience plants, trees, and nature outside of the household (eg, public parks, sports fields, agricultural land, woodlands, coastal paths and nature reserves)’.

Wave 1, specifically designed to assess greenspace utilisation following the implementation of lockdown measures, asked: ‘Have you visited a green space since the movement restrictions have been enforced in the UK? (ie, since 23 March 2020)’. Waves 2–4 inquired whether participants had ‘visited ANY green spaces in the last 4 weeks’. Responses across all waves were categorised binarily as ‘Yes, I have’ or ‘No, I have not’ visited greenspace during the specified time frame.

#### Greenspace use since the COVID-19 pandemic

In wave 4 exclusively, participants self-reported changes in their greenspace utilisation patterns compared with prepandemic behaviour. Participants responded to a question assessing changes in visitation frequency using a five-point scale: ‘Increased a lot’, ‘Increased a little’, ‘No difference’, ‘Decreased a little’, ‘Decreased a lot’ and ‘Don’t know/can’t recall’. For analytical purposes, these responses were subsequently consolidated into four categories: ‘Increased’ (comprising ‘Increased a lot’ and ‘Increased a little’), ‘No change’, ‘Decreased’ (comprising ‘Decreased a little’ and ‘Decreased a lot’) and ‘Don’t know’.

#### Mental health outcomes

Across all four waves, participants evaluated their agreement with the statement: ‘Being in green spaces benefits my mental health (eg, it helps me relax, de-stress)’ based on their ‘own experience in green spaces over the last 4 weeks’. Response options included: ‘Strongly agree’, ‘Somewhat agree’, ‘Neither agree nor disagree’, ‘Somewhat disagree’, ‘Strongly disagree’ and ‘Don’t know/can’t recall’. For analysis, these responses were consolidated into three categories: ‘Agree’ (comprising ‘Strongly agree’ and ‘Somewhat agree’), ‘Other’ (comprising ‘Neither agree nor disagree’, ‘Somewhat disagree’ and ‘Strongly disagree’) and ‘Don’t know’.

### Patient and public involvement

The design of this study was guided and informed by Public Health Scotland’s Environment and Spaces Partnership Group that includes a range of health professionals, civil servants and third sector stakeholders. There was no direct patient or public involvement in the design of this study.

### Analysis

#### Descriptive analysis

The count and proportion of respondents who: (1) visited greenspace within the preceding 4 weeks, (2) reported changes in greenspace utilisation patterns since prepandemic (wave 4 exclusively) and (3) affirmed mental health benefits derived from greenspace exposure are presented. These metrics are stratified by survey wave and further disaggregated by demographic characteristics including age, gender, social grade and ethnicity to identify potential disparities in greenspace engagement and perceived benefits.

#### Statistical analysis

To investigate change in greenspace use over time, as well as change in the reported mental health benefit of spending time in greenspace, both responses were set as outcomes for binary logistic regression models (pooled models including all survey waves). Controlling independent variables were gender, ethnicity and age group; independent variables of interest were wave of survey and social grade. In both models, an interaction between social grade and wave was included to see whether change over time differed between the groups. Where interactions were not significant, they were removed from the final model. P values for interactions were derived from likelihood ratio tests comparing nested models with and without each interaction term (testparm command in Stata); the three-way interaction was tested first, and on finding it non-significant, two-way interactions were each tested in turn. No results were omitted from the analysis and p values are presented in the results. We calculated marginal effects which provide predicted probabilities of the outcomes across different combinations of time points and social grades.

A sensitivity analysis was conducted to assess the impact of residing within an urban (a population of 10 000 people or more) or rural area. Including this variable in the model had no substantive impacts on effect sizes and therefore analysis presented do not include this variable.

To ensure national representativeness, individual level survey weights were applied to adjust for sampling variability across age, geographical region, gender, and social grade distributions. Outcomes are presented at OR, estimated probabilities, and their 95% CIs. The significance level was set at 5%, and all analyses were performed in Stata V.18.

## Results

### Greenspace use in the previous 4 weeks by wave

This study encompassed a total of 8646 respondents, with approximately 2100 participants per wave. Sociodemographic characteristics stratified by wave are presented in online supplemental table 1.

Greenspace utilisation demonstrated variation across survey waves. Peak engagement was observed in the most recent wave (wave 4, April 2025 (86%)), representing postpandemic behaviour. Intermediate and comparable levels were recorded during waves 2 and 3 (64% and 68%, respectively), while the lowest reported use occurred during wave 1 (49%), coinciding with the initial COVID-19 lockdown restrictions (table 1). Consistent socioeconomic gradients in reported greenspace use were evident across all four waves, with individuals of higher social grade reporting greater engagement with greenspace environments. No significant differences in reported use were observed when stratifying by gender or age categories.

**Table 1:** Reported visits to greenspace (GS) in the previous 4 weeks by wave and sociodemographic characteristics

Visited GS in previous 4 weeks	Wave 1–April 20	Wave 2–November 20	Wave 3–April 21	Wave 4–April 25	Total
Total sample					
Yes	1091	1427	1485	1767	5770
%	49.2	65.0	68.3	85.9	66.7
No	1128	769	689	290	2876
%	50.8	35.0	31.7	14.1	33.3
Total	2219	2196	2174	2056	8646
Gender					
Male	510	667	686	824	2688
%	49.6	65.8	67.8	84.4	66.7
Female	581	760	800	935	3076
%	48.8	64.2	68.7	87.3	66.7
Age					
16–24	90	107	124	165	486
%	49.6	61.3	69.5	89.0	67.5
25–64	764	974	1011	1185	3935
%	51.2	65.8	69.2	86.7	67.8
65+	237	349	349	411	1346
%	43.4	64.3	65.3	82.2	63.4
Social grade					
Higher	709	966	972	1090	3738
%	53.1	72.5	73.4	90.5	71.9
Lower	389	475	523	670	2058
%	44.1	54.9	61.4	80.3	59.9
Ethnicity					
White	797	1011	1159	1430	4397
%	47.4	63.9	69.0	85.1	66.4
BAME	44	49	53	348	494
%	44.1	48.9	51.9	89.6	71.5

Number and proportions reported by sociodemographic characteristics are for those who reported visiting GS in the previous 4 weeks.

BAME, Black, Asian and Minority Ethnic.

Ethnic disparities in greenspace utilisation exhibited temporal differences. During wave 1, no significant ethnic differences were detected. However, in waves 2 and 3, individuals of White ethnicity reported higher greenspace engagement. Notably, this pattern reversed in the most recent assessment (wave 4), where participants from BAME backgrounds reported significantly greater greenspace utilisation.

### Mental health benefit of greenspace use

Among participants who reported greenspace visitation within the preceding 4 weeks (n: 5772), almost 9 in 10 agreed that this benefitted their mental health. This perceived benefit remained consistent across waves 2–4 (wave 2: 90%, wave 3: 90%, wave 4: 87%) (table 2). Notably, this proportion was lower for wave 1 (65%), during the initial COVID-19 lockdown restrictions.

**Table 2:** Reported mental health (MH) benefit of greenspace use by wave

MH benefit	Wave 1–April 20	Wave 2–November 20	Wave 3–April 21	Wave 4–April 25	Total
Agree	698	1283	1337	1534	4853
%	65.2	89.5	89.7	86.4	84.1
Other	373	151	154	241	919
%	34.8	10.5	10.3	13.6	15.9
Total	1072	1434	1491	1776	5772

### Self-reported change in greenspace use before COVID-19 compared with the past 4 weeks

Online supplemental table 2 presents comparative data on self-reported greenspace use, contrasting prepandemic behaviour with recent engagement during the preceding 4 weeks, asked exclusively in wave 4 (April 2025). The findings reveal stability in greenspace use for approximately half of the respondents (n=975, 48%), who reported no substantive change in their utilisation patterns. Concurrently, more than one-third of participants (n=766, 38%) indicated an increase in greenspace utilisation compared with prepandemic levels.

### Changes in greenspace use over time by social grade

**Figure 2:**
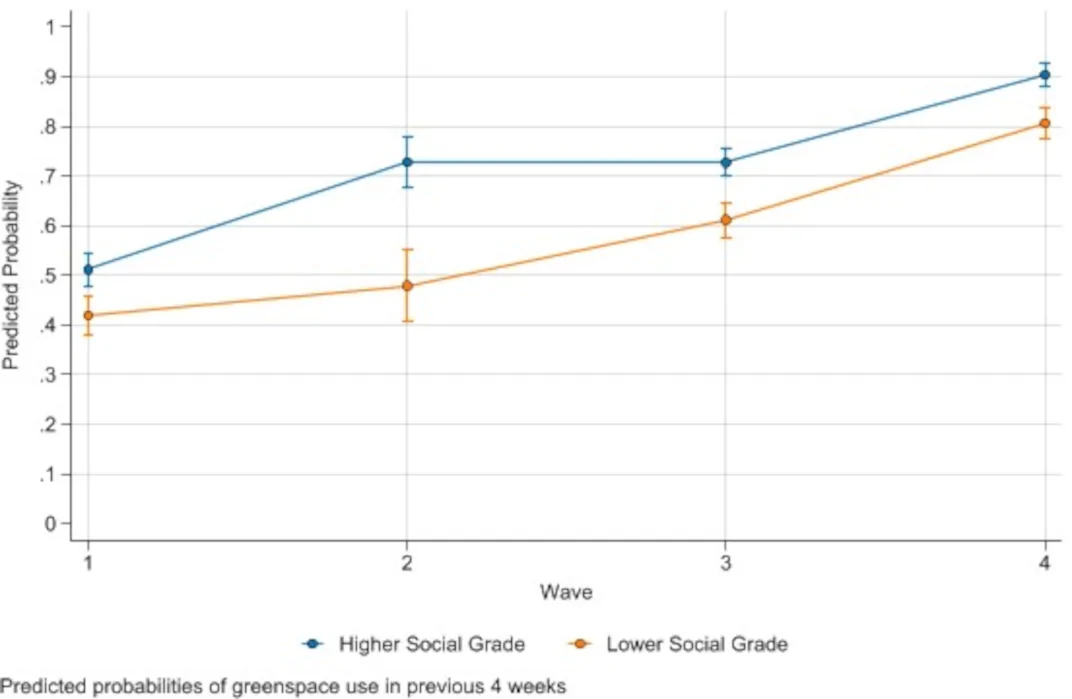
Changes in greenspace use over time by social grade (full model outputs shown in online supplemental table 3).

There was a significant interaction between survey wave and social grade across the whole study period in relation to greenspace visitation patterns (p=0.005), indicating different temporal trends across social grade (online supplemental table 3). Figure 2 illustrates the predicted probabilities of reported greenspace utilisation stratified by social grade across the four survey waves. A persistent socioeconomic gradient in greenspace use was evident throughout the study period, with individuals in higher social grade classifications having greater probability of greenspace use compared with their lower social grade counterparts. However, the probability differential between social grade groups reduced from 25 percentage points in wave 2 to 10 percentage points in wave 4.

#### Change over time in reported mental health benefit of spending time in greenspace and variation by social grade

**Figure 3:**
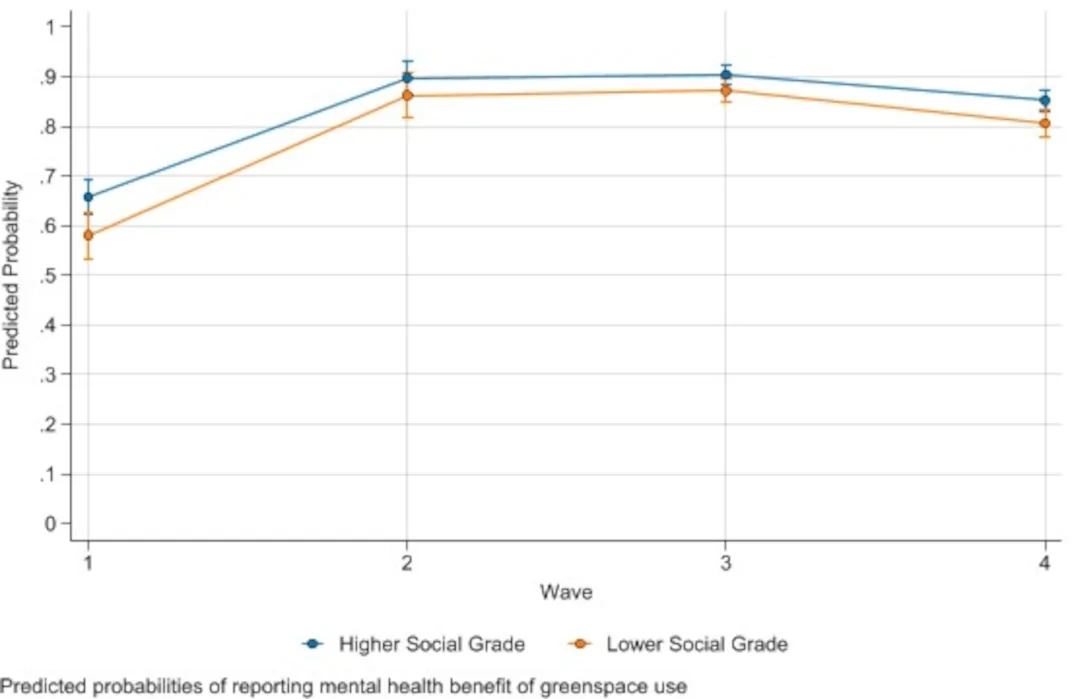
Changes in reported mental health benefit of greenspace use over time by social grade (full model outputs shown in online supplemental table 4).

Analysis of perceived mental health benefit from greenspace use revealed no statistically significant differences between social grade classifications across the survey waves (p=0.194, online supplemental table 4). Figure 3 shows the predicted probabilities of respondents reporting mental health benefit from greenspace use, stratified by social grade across the four measurement waves. An increase in perceived mental health benefits occurred between waves 1 and 2, maintained through wave 3. At wave four the likelihood of agreeing that greenspace benefits mental health declined (p<0.001).

## Discussion

### Summary of findings

This study found a consistent upward trend in greenspace use from April 2020 to April 2025 across a four wave repeat cross-sectional survey of UK adults; use increased from 50% in April 2020 to approximately 66% by April 2021, reaching 85% by April 2025. This finding was corroborated by retrospective self-reports in April 2025, where 38% of respondents indicated increased greenspace engagement compared with prepandemic levels.

Socioeconomic disparities in greenspace use persisted throughout the study period. In wave 4, the predicted probability of greenspace use was 90% for higher social grade participants versus 80% for lower social grade counterparts. Notably, by 2025, greenspace use among lower social grade participants exceeded rates observed in higher social grade groups during the three earlier waves, including the initial pandemic period.

Perceived mental health benefits from greenspace use showed no significant variation by social grade, demonstrating equity in benefit perception across socioeconomic strata. During waves 2 and 3, approximately 89% of greenspace users reported mental health benefits, decreasing slightly to 83% in wave 4. This pattern suggests a substantial and enduring positive shift in greenspace valuation across the population, extending well beyond the acute pandemic phase; however, the recent decrease requires further monitoring.

### Comparison with other literature

Methodological variations necessitate caution when comparing these greenspace use findings with those from other studies and in other settings. Scotland’s People in Nature Survey, employing a distinct metric (outdoor visits for leisure or recreation within a 12-month period), reported 94% of respondents visiting natural spaces at least monthly during 2023/2024.^42^ Similarly, the People and Nature Survey in England found 82% of respondents engaged with green and natural spaces at least monthly between April and June 2024.^43^

Google Mobility data from 2020 to 2022 corroborates our observed trends, documenting a 70% increase in UK greenspace visitations by March 2021 compared with prepandemic baselines.^44^ This followed an initial 30% reduction in April 2020 and preceded a 20% elevation above baseline by November 2021. Global comparisons in the same data for the period February to May 2020 show differences by countries—Italy, Spain and Canada all showing up to 80% decrease in park visitation during March 2020 to early May 2020, which increased up to 80% above baseline by late May 2020.^45^ Countries such as Japan, South Korea and Sweden showed little variation in park visitations during the same time period. Unfortunately, Google Mobility data ceased publication in 2022, precluding comparison with our April 2025 findings.

Environmental factors likely influenced observed utilisation patterns. Research demonstrates positive associations between ambient temperature and greenspace engagement,^44,46^ with adverse weather conditions consistently identified as a barrier to use.^32^ Notably, the UK experienced temperatures 1.7°C above average during wave 4 (April 2025), when greenspace use peaked, contrasting with temperatures 1.7°C below average during wave 3 (April 2021).^47^ During April 2021, the UK average temperature was −1.7°C below baseline.^47^ These weather variations may partially explain fluctuations in reported greenspace use across our survey waves, even though three of the waves took place at similar times of year as higher use was observed during months with higher average temperatures. However, we did not test this within our analysis and propose this as a possible explanatory factor. Future studies should explore the link between longitudinal trends in greenspace use and temperature.

Our findings demonstrate persistent socioeconomic disparities in reported greenspace use despite overall increasing engagement across all social strata. This pattern aligns with other national surveys, including Scotland’s People in Nature Survey, which documented higher participation rates among those ‘very comfortable financially’ (97%) compared with individuals who ‘can only just/can’t afford’ basic necessities (90%).^42^ A study of 33 European cities found that 9 in 10 individuals reported use of forests and parks, which was greater for those with higher incomes, older people and individuals without children.^34^

The data confirm substantial perceived mental health benefits derived from greenspace use, with 87% of users reporting that their recent visits contributed to mental health benefit. The most recent assessment revealed a modest decline of 3 percentage points in perceived benefits. This slight reduction may reflect decreased duration of greenspace engagement per visit (the surveys in our study measured visits rather than duration). Decreased duration aligns with UK Office for National Statistics data indicating diminished leisure time availability compared with 2020–2021.^37^ Previous research established that individuals spending over 120 min weekly in natural environments report significantly enhanced health and well-being outcomes.^48^ Future investigations should concurrently monitor visit frequency, duration and mental health benefits to monitor perceived benefits over time and understand optimal exposure thresholds.

While mental health benefits remained equivalent across socioeconomic strata, lower utilisation rates among lower social grade individuals create population-level disparities in health benefit distribution. Systematic reviews demonstrate that greenspaces often provide greater protective health effects for socioeconomically disadvantaged populations,^49^ suggesting potential equigenic impacts in reducing health inequalities if utilisation disparities were eliminated.^50^ This underscores the importance of targeted multilevel interventions to increase greenspace use among lower socioeconomic groups.

Our results, and others globally,^24^ highlight the importance of greenspace in supporting the mental well-being of residents during the pandemic and beyond. Governments worldwide, including in the UK and European Union, committed to a ‘green pandemic recovery’ which included preserving biodiversity and improving access to greenspaces.^51,52^ However, critical appraisal of policies shows urban developments in Europe are mainly construction-led and as little as 0.3% of spending is allocated to nature-based solutions.^53^ This is further evident in the recent UK Government 2025 Planning and Infrastructure Bill that calls for the review and potential declassification of some protected greenspaces across the country to allow construction on these sites. As national public finances remain strained, it remains important that greenspaces have both investment and protection to ensure they are able to continue to provide their health and well-being benefits.

### Strengths and limitations

This study had a number of strengths. It asked respondents about their greenspace use patterns while evaluating perceived mental health benefits specifically among users, with results stratified by social grade classification. The inclusion of four cross-sectional data waves spanning a 5-year period enabled robust temporal comparisons across the pandemic and postpandemic landscape, providing novel temporal insight which has not been reported. Although data collection involved two distinct survey providers, both employed nationally representative sampling frameworks through UK Omnibus panels with consistent question phrasing, enhancing comparability across waves.

However, important limitations warrant consideration. The repeat cross-sectional design, while valuable for population-level trend analysis, precludes tracking individual-level behavioural changes over time. This limitation constrains our ability to establish causal relationships between greenspace exposure and mental health outcomes.^54^ Additionally, a minor methodological inconsistency exists regarding age inclusion criteria—waves 1–3 sampled adults aged ≥18 years, while wave 4 extended inclusion to those aged ≥16 years. Given the absence of significant differences in reporting patterns between younger age cohorts across waves, all available data were retained to maximise statistical power. There are variations in the ethnic distribution between the four waves, in waves 1 to 3 individuals who reported they were BAME represented approximately 6% of the total sample. For wave 4 this was 18.3%. In the first three waves the BAME population were underrepresented, and it is likely the UK Omnibus survey sampling framework and weighting measures were amended to reflect the 2021 Census for England and Wales (81.7: White; BAME: 18.3%).^55^ Participants were drawn from a panel of individuals who opted to become participants in a national omnibus study. Weighting was applied to ensure national representativeness across age, geographical region, gender and social grade distributions. Physical health limitations or illnesses may influence both the likelihood of visiting greenspaces and mental health outcomes. We were unable to adjust for physical health in our analysis as this information was not collected in wave 4. Future longitudinal studies should consider physical health status as a potential moderator of the relationship between greenspace access, use and mental health benefits. The study includes only one nation and limits its implications for other countries globally. We were unable to assess whether our outcomes varied across specific cities and towns. Evidence shows that both greenspace typologies and their distributions vary geographically across the UK.^56^ Future studies with larger sample sizes should explore whether access to and use of greenspace, as well as associated mental health benefits, differ between major cities, smaller urban areas and rural communities.

## Conclusions

Greenspace use demonstrated robust and sustained growth following the initial pandemic phase, persisting through both 1-year and 5-year follow-up periods. The majority of users consistently acknowledge mental health benefits from greenspace use, though a slight decline observed in the most recent assessment warrants ongoing surveillance and examination. These findings provide crucial evidence supporting greenspace prioritisation in future pandemic preparedness and recovery strategies.

Socioeconomic disparities in greenspace utilisation persist, with lower social grade individuals reporting reduced engagement compared with their higher social grade counterparts, although use has increased for both groups over time. Given that perceived mental health benefits remain equivalent across socioeconomic strata, targeted interventions should address these utilisation inequalities to ensure equitable distribution of greenspace-derived health benefits across all population segments.

Urgent action is required to advance UN Sustainable Development Goal 11, which mandates ‘universal access to safe, inclusive and accessible, green and public spaces’ by 2030. Our findings underscore the importance of improving and protecting greenspaces for healthy and sustainable urban development, while also providing essential evidence for public health surveillance frameworks. Policymakers must prioritise protecting greenspace provision and equitable use to maximise population-level mental health benefits and reduce health inequalities.

## Supplementary material

10.1136/bmjph-2025-003311 online supplemental file 1

## Data Availability

Data are available in a public, open access repository. Data are available on reasonable request.
