# Supplementary material for: Trends in greenspace use, socioeconomic inequalities and mental health benefits following COVID-19 from 2020 to 2025: a four-wave repeat cross-sectional study of UK adults
Source: BMJ Public Health. 2026 Sep 4;4(3):e003311. doi: 10.1136/bmjph-2025-003311 (PMC13548254; doi:10.1136/bmjph-2025-003311)
Supplement: Supplementary file 1 [file bmjph-4-3-s001.docx]

**Trends in greenspace use, socioeconomic inequalities and mental health benefits following COVID-19 from 2020 to 2025: A four-wave repeat cross-sectional study of UK adults.**

**Supplementary Materials**

Supplementary Table 1: Participant characteristics.

|  | **Wave 1 – Apr 20** | **Wave 2 – Nov 20** | **Wave 3 – Apr 21** | **Wave 4 – Apr 25** | **Total** |
| --- | --- | --- | --- | --- | --- |
| **Gender** | | | | | |
| Male | 1,093 | 1,090 | 1,075 | 1,039 | 4,296 |
| *%* | *48.5* | *48.5* | *48.5* | *48.8* | *48.6* |
| Female | 1,160 | 1,157 | 1,141 | 1,090 | 4,548 |
| *%* | *51.5* | *51.5* | *51.5* | *51.2* | *51.4* |
| **Age** | | | | | |
| 16 to 24 | 250 | 249 | 246 | 260 | 1,005 |
| *%* | *11.1* | *11.1* | *11.1* | *12.2* | *11.4* |
| 25 to 64 | 1,482 | 1,478 | 1,457 | 1,389 | 5,806 |
| *%* | *65.8* | *65.8* | *65.8* | *65.0* | *65.6* |
| 65+ | 520 | 519 | 512 | 488 | 2,038 |
| *%* | *23.1* | *23.1* | *23.1* | *22.8* | *23.0* |
| **Social grade** | | | | | |
| Lower | 1,284 | 1,281 | 1,263 | 1,175 | 5,003 |
| *%* | *57.0* | *57.0* | *57.0* | *55.4* | *56.6* |
| Higher | 969 | 966 | 953 | 944 | 3,832 |
| *%* | *43.0* | *43.0* | *43.0* | *44.6* | *43.4* |
| **Ethnicity** | | | | | |
| White | 1,703 | 1,615 | 1,718 | 1,746 | 6,783 |
| *%* | *94.1* | *94.0* | *94.4* | *81.7* | *90.6* |
| BAME | 106 | 103 | 102 | 390 | 701 |
| *%* | *5.9* | *6.0* | *5.6* | *18.3* | *9.4* |
| Total | 2,219 | 2,196 | 2,174 | 2,056 | 8,646 |

Supplementary Table 2: Change in Greenspace Use before the Covid-19 pandemic compared to the past 4 weeks.

| **Change in GS use after covid** | **Freq** | **%** |
| --- | --- | --- |
| Increased | 766 | 38 |
| No change | 975 | 48 |
| Decrease | 299 | 15 |
| Total | 2,040 | 100 |

Supplementary Table 3: Likelihood of Greenspace Use by wave and social grade (interaction and marginal effects).

| **Visited GS in previous 4 weeks** | **Odds ratio** | **LL 95 % CI** | **UL 95 % CI** | **P value** |
| --- | --- | --- | --- | --- |
| **Gender** | | | | |
| Male | *reference group* | | | |
| Female | 1.02 | 0.90 | 1.16 | 0.74 |
| **Age** | | | | |
| 16 to 24 | *reference group* | | | |
| 25 to 34 | 1.24 | 0.91 | 1.69 | 0.18 |
| 35 to 44 | 0.98 | 0.72 | 1.33 | 0.90 |
| 45 to 54 | 1.01 | 0.75 | 1.36 | 0.96 |
| 55 to 64 | 0.96 | 0.71 | 1.30 | 0.78 |
| 65+ | 0.88 | 0.67 | 1.15 | 0.35 |
| **Ethnicity** | | | | |
| White | *reference group* | | | |
| BAME | 0.77 | 0.59 | 0.99 | 0.05 |
| **Wave** | | | | |
| Wave 1 | *reference group* | | | |
| Wave 2 | 2.56 | 1.92 | 3.41 | <0.001 |
| Wave 3 | 2.56 | 2.11 | 3.09 | <0.001 |
| Wave 4 | 9.00 | 6.69 | 12.11 | <0.001 |
| **Social Grade** | | | | |
| Higher | *reference group* | | | |
| Lower | 0.69 | 0.56 | 0.85 | <0.001 |
| **Interaction: Wave # Social Grade** | | | | |
| 2#Lower | 0.50 | 0.33 | 0.76 | <0.001 |
| 3#Lower | 0.85 | 0.64 | 1.14 | 0.29 |
| 4#Lower | 0.64 | 0.44 | 0.95 | 0.03 |
| **Marginal effects** | | | | |
| **(Wave # Social Grade)** | | **Predicted probability** | **LL 95 % CI** | **UL 95 % CI** |
| 1#Higher | | 0.51 | 0.48 | 0.54 |
| 1#Lower | | 0.42 | 0.38 | 0.46 |
| 2#Higher | | 0.73 | 0.68 | 0.78 |
| 2#Lower | | 0.48 | 0.41 | 0.55 |
| 3#Higher | | 0.73 | 0.70 | 0.76 |
| 3#Lower | | 0.61 | 0.57 | 0.65 |
| 4#Higher | | 0.90 | 0.88 | 0.93 |
| 4#Lower | | 0.81 | 0.78 | 0.84 |

Supplementary Table 4: Likelihood of mental health benefit for greenspace users by wave and social grade (interaction and marginal effects).

| **MH benefit** | **Odds ratio** | **LL 95 % CI** | **UL 95 % CI** | **P value** |
| --- | --- | --- | --- | --- |
| **Gender** | | | | |
| Male | *reference group* | | | |
| Female | 1.25 | 1.05 | 1.49 | 0.01 |
| **Age** | | | | |
| 16 to 24 | *reference group* | | | |
| 25 to 34 | 1.79 | 1.22 | 2.62 | <0.001 |
| 35 to 44 | 1.34 | 0.92 | 1.96 | 0.13 |
| 45 to 54 | 1.41 | 0.96 | 2.06 | 0.08 |
| 55 to 64 | 1.50 | 1.03 | 2.19 | 0.04 |
| 65+ | 1.32 | 0.94 | 1.85 | 0.12 |
| **Ethnicity** | | | | |
| White | *reference group* | | | |
| BAME | 0.86 | 0.63 | 1.17 | 0.34 |
| **Wave** | | | | |
| Wave 1 | *reference group* | | | |
| Wave 2 | 4.23 | 2.63 | 6.79 | <0.001 |
| Wave 3 | 4.37 | 3.21 | 5.96 | <0.001 |
| Wave 4 | 3.13 | 2.36 | 4.16 | <0.001 |
| **Social Grade** | | | | |
| Higher | *reference group* | | | |
| Lower | 0.69 | 0.51 | 0.93 | 0.02 |
| **Interaction: Wave # Social Grade** | | | | |
| 2#Lower | 1.22 | 0.57 | 2.63 | 0.61 |
| 3#Lower | 1.36 | 0.83 | 2.23 | 0.23 |
| 4#Lower | 0.95 | 0.62 | 1.44 | 0.80 |
| **Marginal effects** | | | | |
| **(Wave # Social Grade)** | | **Predicted probability** | **LL 95 % CI** | **UL 95 % CI** |
| 1#Higher | | 0.66 | 0.62 | 0.69 |
| 1#Lower | | 0.58 | 0.53 | 0.63 |
| 2#Higher | | 0.90 | 0.86 | 0.93 |
| 2#Lower | | 0.86 | 0.82 | 0.91 |
| 3#Higher | | 0.90 | 0.89 | 0.92 |
| 3#Lower | | 0.87 | 0.85 | 0.90 |
| 4#Higher | | 0.85 | 0.83 | 0.87 |
| 4#Lower | | 0.81 | 0.78 | 0.83 |
